# A qualitative study of health care access among African American older adults in a socio-demographically under-resourced region during the COVID-19 pandemic

**DOI:** 10.3389/fpubh.2022.893718

**Published:** 2022-11-24

**Authors:** Lucy Annang Ingram, Cheryl Dye, Heather Boger, Ye Luo, Tara Hayes

**Affiliations:** ^1^Department of Health Promotion, Education and Behavior, University of South Carolina, Columbia, SC, United States; ^2^Department of Health Promotion, Education, and Behavior, Arnold School of Public Health, University of South Carolina, Columbia, SC, United States; ^3^Institute for Engaged Aging, Clemson University, Clemson, SC, United States; ^4^Department of Neuroscience, Medical University of South Carolina, Charleston, SC, United States

**Keywords:** health care access, African American, COVID-19, older adult, health disparities

## Abstract

**Background:**

In the U.S., health inequities experienced by the African American community, specifically among those ages 65 and older, have been well-documented in research literature. Alongside the findings regarding disparities in disease prevalence and management, researchers have also highlighted disparities in health care access. Despite recent evidence of health inequities experienced by African Americans during the COVID pandemic, there is little research on the lived experience of this group in this critical time, health care access challenges that may be exacerbated by the pandemic, and the community's outlook for the future in addressing health disparities.

**Methods:**

We conducted a qualitative study of African Americans to gather their perspectives about access to health care, particularly during the COVID-19 pandemic. Study participants consisted of African Americans, ages 50–85 years, who spoke English as their primary language, who resided in one of 17 counties in South Carolina that represent a region of the State known as the corridor of economic disadvantage.

**Results:**

Forty-seven telephone interviews were conducted. While research has shown that certain populations experienced health care access disparities during the early COVID pandemic, these disparities did not appear to be exacerbated in our sample. However, participants noted an increase in the use of telehealth, and identified challenges to using this technology. Participants made recommendations about how to address disparities in health care access in their communities.

**Conclusion:**

Our qualitative approach was useful in obtaining perspectives about access to health care during the COVID-19 pandemic from African American older adults. Continued research with older African Americans, particularly those in under-resourced communities are warranted to further elucidate these findings.

## Introduction

In the U.S., health inequities among African Americans, specifically among those ages 65 and older, have been well documented in research literature. For instance, African Americans have higher rates of chronic disease such as high LDL cholesterol, heart disease, and diabetes (1–3) compared to others of different racial and ethnic backgrounds. These preexisting conditions are more saturated among, and less likely to be effectively managed by, African Americans (2, 4) compared to other racial and ethnic groups. This has resulted in higher death tolls, lower quality of life, and lower life expectancies for older African Americans (5). The most enduring primary contributing factor to the avoidable differences in health outcomes experienced by this community is socioeconomic status.

Alongside the findings regarding disparities in disease prevalence and management, research has also revealed disparities in health care access (2, 6). The causes of health care access disparities are multifaceted and include social and economically driven factors such as intersectionality, health insurance coverage and affordability, geography, educational level, housing conditions, and lack of adequate guidance about how to navigate the health care system (7). According to the growing body of literature that has emerged since the start of the COVID-19 pandemic, many health care disparities have been reinforced, and others have become even more substantial (8–10). The sections that follow briefly review the literature on intersectionality and health insurance coverage to delineate some of the challenges in health care access among African Americans.

### Intersectionality

Intersectionality helps in understanding how “multiple social identities…intersect to reflect interlocking systems of privilege and oppression at the macro social-structural level” (11). In this instance, identifying as both an older adult and an African American are the relevant social identities. These characteristics have been two of the most salient risk factors for contracting COVID-19 in the U.S. According to the CDC, persons aged 65 and older have a greater chance of both contracting and dying from the virus compared to other age groups. Over 81% of COVID-19 deaths are associated with persons ages 65 and older with preexisting conditions (12). Furthermore, African Americans not only contract the virus at more alarming rates, but also suffer more negative health outcomes from the pandemic compared to their White counterparts (3). African Americans suffer hospitalization and death at almost double the rates of persons of other racial groups (13).

Three factors have been found to contribute to adverse health outcomes in the older African American population during the COVID-19 pandemic: risk of exposure, the weathering processes, and health care access and quality. This population has a higher risk of exposure due to living in multigenerational households and their employment conditions (e.g., jobs that frequently interface with the public). Over 41% of African Americans work front-line jobs in low-income and under-resourced neighborhoods. This population also has high instances of older family members living within the home, increasing exposure risks (14). Weathering processes refer to the phenomenon of premature biological aging due to exposure to social adversity such as racism (15). Racism-related stressors are correlated with increased activity in the sympathetic nervous symptom, a finding which is correlated with higher instances of increased blood pressure in African Americans (15). Lastly, research has shown that inequality in health care access and quality further exacerbate African American health and wellbeing (2, 6, 16–18). Addressing these inequities will help to mitigate the impact of COVID-19 on marginalized groups.

### Health insurance coverage

According to 2019 South Carolina Behavioral Risk Factor Surveillance Survey (SCBRFSS) data collected from those aged 50 years and above, non-Hispanic White persons were more likely to report health care coverage (94 vs. 90%) and that they had one person they thought of as their personal health care provider (92 vs. 89%) compared to non-Hispanic Black persons. White persons were less likely to report that they needed to see a doctor in the past 12 months but could not because of cost (8.8 vs. 14%) compared to Black persons. However, non-Hispanic Black persons were more likely to report they had received a checkup from a doctor in the past 12 months (93.8%) compared to non-Hispanic White persons (89.2%) (19). When considering the additional impact of COVID-19, economic hardship, co-morbidities, and challenges with visiting health care providers (e.g., shuttered doors, fear of contracting COVID, telehealth inconsistencies, etc…) worsened access to care for older adults, particularly African Americans, and persons with geographic barriers that made getting to the doctor difficult (15, 20, 21).

Despite recent evidence of health inequities experienced by African Americans during the COVID pandemic, there is little research on the lived experience of this group in this critical time, any health care access challenges that may be exacerbated by the pandemic, and the community's outlook for the future in addressing health disparities. Research on the extent of testing and vaccine disparity has been rich; however, research regarding the community's perspective about fundamental causes of disparities has been limited (18). To address this gap, we conducted a qualitative study of older African American adults to gather their perspectives about access to health care, particularly during the COVID-19 pandemic.

## Method

### Study Participants

The rigor of qualitative methods such as those used in our study has been well established (22, 23). The key principles of qualitative analysis promote the use of systematic methods to ensure rigor and quality of the approach and thus validity and meaningfulness of study findings. As described below, we adhered to these principles in our study. We conducted interviews with African American older adults about factors that contribute to health disparities, their perceptions of selected health disparities, and potential strategies to address the disparities. A particular focus of these interviews was given to participants' perspectives about access to health care during the COVID pandemic which is the focus of this paper. Study participants consisted of African Americans, ages 50–uth Carolina that represent a region of the State known as the corridor of economic disadvantage. This rural and underserved region of the State has historically been poorly resourced, is economically distressed (i.e., low income, high unemployment, and high poverty), and_has under-resourced schools resulting in low educational attainment. This toxic combination of factors yields significant health disparities among this population.

### Recruitment strategy

A local marketing research group managed participant recruitment. In order to access the population, an e-mail blast was distributed to over 180 African Americans in the firm's databases whose profiles suggested they lived in one of the target counties and met the age criteria. Social media ads and posts were also used. For example, Facebook ads were posted seeking South Carolina African Americans who resided in specific counties, and were ages 50–85 years, for a paid telephone research project. One of these posts had a paid boost that was concentrated in Hampton, Jasper, and Allendale counties (specifically targeting social media users in these areas). Requests for participants were also posted in targeted Facebook affinity groups (i.e., Hampton County SC Buy Sell Trade Wanted, Marion County Habitat for Humanity, Lowcountry Government Facebook, and For Sale, Wanted, or Free in Darlington SC). Additionally, the recruiters reached out to several different agencies and organizations in specific counties to solicit assistance in identifying potentially eligible respondents, particularly those who did not complete high school or a GED. Flyers were posted in key areas in these locations, and local staff was offered an incentive for referring older African Americans who qualified and ultimately completed the telephone interview.

### Instrumentation

As part of the screening process, participants were asked demographic questions about their age, sex, income level and education level, and county of residence to determine their eligibility. Also, an interview guide was developed to standardize the conduct of interviews. Open-ended questions were developed by the research team led by the first author based on the research aims of the study. The questions were guided by a review of extant literature that had been led by the second author regarding common health disparities experienced by African American older adults including chronic disease, obesity, and dental health. Additional questions focused on COVID experience and impact, namely telehealth, social isolation, and vaccine hesitancy. Additionally, questions were adapted from existing validated instruments such as the Centers for Disease Control and Prevention (CDC) Behavioral Risk Factor Surveillance System (BRFSS), the CDC Household Pulse Survey, and the National Social Life, Health, and Aging Project (NSHAP). The interview questions were pre-tested with members of the intended population to ensure content validity. Based on the feedback received during the pre-test, the interview questions were refined and finalized. Such refinements included re-wording questions to ensure face validity and removing questions that did not fit within the scope of the research. The data presented here will focus on participant responses to questions about COVID-19 and its impact on health care access.

### Procedure

Prospective participants were invited to participate in a telephone interview about racial differences in health among the 50 and older African American population in South Carolina. Staff trained in proper conduct of interviews and protection of human subjects research served as interviewers. Interviews were scheduled for days and times that were convenient to study participants. All participants received a $100 incentive for their participation. The telephone interviews were conducted over a nine-week period, from May through July 2021. Interviews lasted on average about 45 min each.

### Data Analysis

Interviews were digitally audio-recorded, and audio recordings were transcribed for review and analysis. The analysis team consisted of a primary and secondary coder, with collective research expertise in the following areas: racial health disparities, African American health, health promotion, chronic disease self-management, disease prevention, and older adult health. Both coders were responsible for reviewing and coding transcripts independently, that is, each transcript was independently reviewed by each coder. Further, the primary coder was responsible for developing the initial codebook and establishing a sound basis for the themes that were included. The coding process began with pre-constructed codes with additional codes added as part of an iterative process. The establishment of a priori themes was guided by a comprehensive review of the literature that focused on the leading health disparities experienced by African Americans, with particular interest in the health disparities experienced by African Americans during the height of COVID-19. For example, one a priori theme was *telehealth convenience during COVID*. An emergent theme was the *impersonality of telehealth*. Another example of an a priori theme was the concern about *complications of the COVID-19 vaccine*. An emergent theme was about the *ease of access in obtaining the vaccine* (an unexpected theme given the media attention at the time of data collection regarding challenges to vaccine access). Codes were added during the transcript review process until the coders agreed that the review had yielded saturation. Any disagreements were discussed until the team reached consensus about the final codes to be included in the process of establishing the codebook. This approach is a method that has been used widely in other qualitative analyses (22).

## Results

### Characteristics of the sample

Forty-seven telephone interviews were conducted with African Americans ages 50 and older in South Carolina. The majority (57.7%) were female, 51.0% reported having an annual income <200% of the poverty level, and nearly 60% had greater than a high school diploma or GED. Participants resided in one of 17 counties in South Carolina that represent areas of socio-economic disadvantage within the State. See Table 1 for the demographic characteristics of the sample.

**Table 1:** Demographic characteristics of the sample (*N* = 47).

**Characteristic**	***N* (%)**
**Age**	
50–59	17 (37.7)
60–69	15 (33.3)
70–79	13 (28.8)
80–85	2 (4.4)
**Sex**	
Male	20 (42.5)
Female	25 (57.5)
**Income**	
Income <200% poverty	24 (51.0)
Income > 200% poverty	23 (49.0)
**Highest level of education**	
Less than high school graduate/GED	16 (34.0)
High school/GED	3 (6.4)
Greater than high school/GED	28 (59.6)
**County of residence**	
Allendale	2 (4.3)
Bamberg	1 (2.1)
Beaufort	4 (8.5)
Clarendon	1 (2.1)
Colleton	4 (8.5)
Darlington	3 (6.4)
Dillon	1 (2.1)
Dorchester	4 (8.5)
Florence	5 (10.6)
Hampton	1 (2.1)
Jasper	1 (2.1)
Lee	3 (6.3)
Marion	2 (4.3)
Marlboro	1 (2.1)
Orangeburg	5 (10.6)
Sumter	5 (10.6)
Williamsburg	4 (8.5)

### Qualitative findings

Below is a summary of themes from the participant interviews highlighting responses to questions related to COVID-19 and its impact on health care access. Representative quotes are included to help elucidate findings, and each quote is referenced by a pseudonym (fictitious name used to protect participants' anonymity). The themes presented represent the most frequently noted phenomena by the participants.

#### COVID-19 impact on health care access

Participants were asked about the impact of COVID-19 on their access to health care. Participants noted that the primary impact of the pandemic on their ability to access health care was in the form of re-scheduled appointments by their providers. One participant stated that, “*a lot of my appointments [were] canceled by the physician.”* [Steven] Another participant noted, “*I avoid going to the doctor because for one thing, the doctors, they wasn't letting you come to their office anyway. So, I just took my medicine like I was supposed to take it.”* [Teresa].

Conversely, many participants offered that they felt no impact by the pandemic on their ability to access health services. As noted by Sandra: “*I kept all my doctor's appointments. I took my shots. I did whatever needed to be done to keep myself safe.”* As stated by another: “*Well actually, I would try to keep up with my medical care. If I had needed to go, just say if I was out of a prescription and I was just going to the doctor, going like I should, I wouldn't have to go in because they could call in a prescription…But at first, I didn't want to be out around in places like that around a lot of people. And I didn't.”* [Cynthia].

#### Telehealth experience

Given some of the accommodations that were made to address the need to social distance to mitigate the spread of COVID-19, many health centers shifted to offering telehealth services to still meet the demands of some of their patient population. Participants were asked about their experience with telehealth and any suggestions they may have to enhance telehealth services. Themes that emerged regarding telehealth experience were: impersonality of the services and increased convenience. Themes regarding solutions to enhance telehealth included: providing instructions to older adults and addressing Internet barriers.

Regarding an impersonal telehealth experience, participants noted missing the human connection that a face-to-face office visit provides. For example, one participant noted: “*She was really nice, but personally, I do not like this over-the-phone conference or whatever you want to call it with doctors, I do not. If I've got pain in my chest, how can you diagnose my pain in my chest or know the rhythm of my heart, I'm having a heart attack or whatever, just looking at me over the phone?”* [Teresa]. As stated by another participant: “*It's the only reason I had the virtual, but I just didn't like the idea because it's not hands-on and it's not my doctor that I normally see. If I had any questions I could ask him there at the facility, something I might be going through. And then I couldn't get my urine test and my blood test like I normally do.”* [Robert].

Alternatively, some participants remarked about the convenience of the telehealth services, primarily “*because you didn't have to leave home for number one.”* [Larry]. According to Karen: “*It worked out well. It was great because I didn't have to wait for an appointment, I could get what I needed right away. I think it's great. It's great. Yes.”*

Participants also suggested solutions to enhance telehealth services. The first theme that emerged was to provide instruction and opportunities to practice for older adults. As noted by Sandra: “*For me, it will be hard because I never did and I don't know how to. I know somewhat how to get on the Internet, but I never did it.”* Further, Denise remarked: “*You're going to have to really get some sort of an outreach program, to go in or have them come and so they can be taught. I mean, it's just the same thing like a smartphone, getting my mom to understand how that works. You need someone to sit down and explain that to them.”*

Lastly, another theme that emerged regarding potential solutions was to address Internet challenges. One participant stated about accessing telehealth: “*It would be hard because my parents don't have Internet.”* [Donald]. According to another: “*A lot of people still don't have Internet. A lot of Black folks still don't have Internet or still don't have access to computers. So, that would be one.”* [Bruce].

#### Perspectives on the COVID-19 vaccine

There are a variety of perspectives about the COVID-19 vaccine, some of which yield what has been termed “vaccine hesitancy,” particularly among people of color. Given this, this particular group of questions was of great importance. Themes that emerged regarding participants' perspectives on the vaccine included: ease of obtaining the vaccine, skepticism about the vaccine, and concerns about potential complications from the vaccine.

Several participants described from either first-hand experience or from others that obtained the COVID-19 vaccine, that it was an easy process. As noted by Charles: “*Everybody around here says it was easy to get when it was time for them.”* Denise offered a similar sentiment stating: “*It was easy. I signed her up and I went and took her to the first one, and I took her to the second one. We stayed in the car and they came and did it there, and that was it.”* Steven stated: “*It wasn't no big deal. They said it was here and they went, my father-in-law, he went straight and got his. Everybody I knew that was older than me, they didn't have no... They didn't have no bunch of red tape to go through.”*

While there was much discussion about the ease of accessing the vaccine, there were several comments about skepticism regarding the vaccine. Some of the basis for the skepticism was steeped in historical events, others with personal experience, and others based on current events. For example, Gary stated: “*…they find a vaccine that would slow it up. Wasn't no guarantee that it would stop it. But it slows it up. But Black people would kind of hesitate because what happened many years ago, long years ago. We heard that they used to use Black people as the guinea pigs as far as…any kind of experiment.”* Richard offered his own personal experience with shifts in skepticism. He states:

*We've had the Moderna. In the beginning, during the Trump era, I was a little nervous and it had nothing to do with the President. My question was, if we can't get a vaccine for AIDS, which is something that's been out a long time, how were they able to get this so fast? So, I was a little nervous, then when I started seeing it on TV. I started seeing a lot of African-Americans on TV saying they've got it. So, I've said, “Okay. Well then, some of them got it.” Then some people I know had told me, “Listen, we went and got it. It was okay. I think you should get it.” So, by the time for me to get it, I was a little more at ease*.

Gary also discussed how the debate about which vaccine to take as well as reports of issues fueled his skepticism:

*After you keep hearing different things about it, they had a call back and something happened about one or two times with Johnson. That scared me right then. So, I went to Moderna. I take the other one. I already had an appointment and everything with Johnson. But I changed my mind because of the things that we heard about Johnson*.

Another source of concern was about the potential complications and side effects from the vaccine. For instance, Denise was concerned that “*maybe it won't act right with the medications that I'm taking.”* Similarly, Cynthia states this concern: “*I guess sometimes I think about what about the side effects, which some people don't have no side effects but some do. And then I read that some have had the vaccine and shortly after they had it, a couple days, they died.”* Another comment was in regard to blood clotting: “*I want to wait and see the results from other people first. I want to see, because I hear people getting blood clots and different things. So, I basically want to wait, just for about six months.”* [Karen].

Participants were asked for their suggestions about how to improve vaccination rates among African Americans ages 50 and older. Themes that emerged included: providing more information about the vaccine, engaging community organizations to provide information, and using familiar faces/trusted person as sources of information about the vaccine.

Related to providing more information, participants suggested that African Americans, in particular, need more information in order to make informed decisions about getting the vaccine. Denise remarked about the need to accommodate the lack of trust among some African Americans. She stated: “*I think that it should be more information given because of us here, especially, not being trusting. We're not too trusting because there's been so much that happened here in South Carolina, with us being given stuff because of us being African-Americans, bad stuff.”* Patricia suggested the type of information that should be provided. She stated: “*I think everything's being done, but I think there needs to be more education about it because what I'm hearing is the whole premise is, get the vaccination, get the vaccination, get the vaccination, but really educating and telling them why and the importance of getting it.”* Likewise, Kenneth stated: “*I think if they stress it more and telling more and more, and that it won't be that bad on you if you get a shot done. Because I kept saying I wasn't, then I talked about it and I had it. Even though it made me feel bad, I went through a rough day afterwards, but I'm glad I took it.”*

Others suggested that community organizations would be ideal sources to disseminate information about the vaccine and help address concerns. As noted by Robert: “*And I think now I believe the community leaders and the church pastors are now getting involved with it so I think little by little, I think the process of the African American getting that shot, is going to pick up tremendously.”* Similarly, Charles stated: “*More information at the churches and senior citizen groups. If you can provide information to them, like the little senior citizen clubs, where they are just sitting around talking, if there is some kind of way you can have pamphlets or someone to have discussion.” One* participant specifically noted that this approach is critical in rural communities. Cheryl stated: “*In the rural African American communities, you have to get stuff to the church. You have to get it to the church and you have to get it to the members or somebody within the community that interacts with the people within the community.”*

Lastly, an important strategy that was noted was to use familiar faces and trusted persons as persons to deliver information about the vaccine. For example, Teresa stated: “*I read for myself, read up on different stuff. That helps me. And, maybe for other people, maybe if they had someone that they trust talk to them about it. Maybe their children might can convince [them] or someone that already had it. That might help…”* Similarly, Steven noted: “*Get somebody or you identify somebody out of that area that they trust. That they know personally. Oh, I know him. He's straight up. I'll do it. But if they send a bunch of strangers, no, that ain't going to work.”*

The final set of questions was about organizational partnerships and their effectiveness in promoting health and access to health care among persons in their community. Emergent themes included: having more attentive physicians, promoting fair medical treatment, and increased advocacy and family support.

Participants noted that an important factor for promoting health and health care access by community partners is to have more attentive physicians. Some of that, begins with trust. Robert stated: “*You almost have to start early where we could trust doctors.”* Charles recounts his own experience with an attentive physician: “*She's more attentive to your health. She would go over your chart and if it was time for you to have a blood test for this or a blood test for that, or an EKG or a scan…she was more attentive to you. Like you were the only patient that she had. We need more doctors like that.”* Cynthia also describes the qualities of an attentive physician. She noted:

“*Well, explain to them what's what. Don't rush them whenever you go to an appointment. Are you asking questions, but then you rush and you know that you're being rushed. Sometimes some doctors act like they don't have time to explain something to them, but then they want to write you a prescription for this. But then, they don't have all their questions answered. So, that brings up a flag as well, too.”*

Partnering with organizations that promote fair medical treatment was also a suggested strategy for promoting health care. For instance, Bruce and Denise remarked about fair treatment respective of insurance coverage. Bruce stated: “*Just because your insurance is a better insurance than mine, that shouldn't make a difference. I should get the same treatment that you get.”* As noted by Denise: “*They need to do more. They need to look at each particular State in detail and see what's happening here. It's ridiculous to work all your life and then get sick, and you don't have any extra help because they just don't allow you.”* Trust was also a factor that contributed to fair treatment. As explained by Cheryl:

“*In order for African Americans to have that trust, it has to be doctors within a facility that they have access to, that they know that there are doctors that they can trust, they know that they can go to those doctors and not be judged that, I know they're not going to do right because as soon as they see the color of your skin they don't treat you right or they don't do right.”*

Lastly, having supportive family or other advocates who can intercede when needed was noted as an important factor to promote health. Charles stated: “*I was trying to think of a group, but I was thinking more family. Younger family should keep an eye out for older family and stress upon them that they do need to go to the doctor.”* Cynthia describes non-familial advocate support in this way:

*Well, with some insurance, they do have things like where the nurse or someone will come out, ask them about their medications and this and that. It is some things out there like that, but some don't take it up…And some, when they go to the doctor, they don't really know about a lot of things going on with them. Like I say, they could have a family member or their health care provider to go with them*.

Gary suggests that there is much support available, but some are unaware. According to him: “*I think they should find out more about what's available because a lot of time people don't keep up with the news or the best way of going about getting help. Because it's a lot of help out there, but you got to know who to talk to and where to go.”*

## Discussion

While research has shown that certain populations experienced health care access disparities during the early COVID pandemic (8–10), these disparities did not appear to be exacerbated in our sample. Based on our findings, participants identified few disruptions in routine health care access due to COVID-19. Many reported either re-scheduled health care visits or no impact of the pandemic on their access. Of note is that, in communities where access may already be problematic, as long as the situation doesn't worsen, community members may remain satisfied with the status quo. An opportunity to capture a true baseline measure of health care access prior to the pandemic would further elucidate this finding.

Participants did report disparagingly about their telehealth experiences, noting primarily that the experiences felt impersonal, and that common challenges included lack of instruction about how to use the technology and barriers to Internet access. Recent research from some scholars has documented African Americans using telehealth increasingly over the course of the pandemic; however, they do not report input from the community about the barriers to telehealth access that were experienced at the height of the pandemic (24). The effects of public compared to private insurance on telehealth use were also recently documented by Schenker (25). Individuals with public health insurance, such as that found in the Affordable Care Act (ACA) expansion and widely held by the older African American community, used telehealth at much lower rates than those with private health insurance. In order for telehealth to work effectively, a patient must have reliable access to both technology and broadband service (26). These resources are not widely available in older, racial and ethnic minority populations, creating a disparity in telehealth experience for older African Americans (27). Older populations are also at a specific disadvantage of lower rates of technology knowledge and slower technology adaptation (28). By increasing access to affordable technology and Internet providers, older African Americans can have positive telehealth experiences, particularly those in rural communities (26, 29).

Regarding the COVID-19 vaccine, we found a range of perspectives from ease of access, to skepticism and concern about complications from the vaccine. Vaccine hesitancy is defined as uncertainty and ambivalence about vaccination (30). Nationwide studies have shown that Whites are receiving the COVID vaccine at two times higher rates than African Americans (13% of Whites vs. 7% of African Americans) (31, 32). The National Association for the Advancement of Colored People (NAACP) and its partners also conducted a survey that reported that only 14% of African Americans trusted the vaccines' safety and only 18% said they would like to be vaccinated (33). Contrary to national statistics, African Americans in South Carolina currently account for 58.51% of completed vaccine distributions, while Whites account for 22.94% (34). The noted differences in vaccination rates across the State can be explained by geographic challenges to accessing health care in rural areas. Rural counties in South Carolina are experiencing up to 20% lower vaccination rates than the State's overall average of 55% (34).

Further, our participants attributed vaccine hesitancy among African Americans to both historical references to maltreatment of African Americans in the U.S. and distrust of the medical community. One of the foremost examples of unethical medical treatment of African Americans is the U.S. Public Health Service Syphilis Study at Tuskegee. This study provides important context for understanding African Americans' loss of confidence in the U.S. health care system (35, 36). As a result of the medical experimentation on 600 poor African American males in rural Alabama that occurred 1932–1972, the health of the men in the research study was compromised, men's lives were lost, and their families suffered the fate of these men as well as their own residual physical and mental health challenges. Some researchers suggest that African Americans possess a communal medical distrust (37), a position that should not be surprising given the harrowing history of African Americans' engagement with the U.S. health care system. During the pandemic, this distrust has taken the form of what the general public calls “conspiracy beliefs” about the virus. Bogart et al. (38) found that 34% of African Americans believed that COVID-19 was a government-fabricated virus, created in a lab, to encourage vaccinations. This is in comparison to 26% of their White counterparts. The Tuskegee Study is but one example that represents countless other instances of experimentation and exploitation on African Americans and is a meaningful indicator of the work that needs to be done to build and sustain trusting relationships between African Americans, particularly those in the American South, and the health care system.

An important corollary to the discussion of vaccine hesitancy is recognition of the systems and structures that perpetuate this sentiment. Dr. Vanessa Northington Gamble, internationally renowned expert on the history of American medicine and its intersection with racial and ethnic disparities and bioethics, states: “I think the problem is, with this focus on the distrust of African Americans, it becomes like this inherent trait of African Americans, that these distrustful Black people, as opposed to focusing on a health care system that does not have trustworthiness” (37). This perspective was echoed by our study participants and is a significant indicator of the work to be done to make substantial improvements to the U.S. health care system.

Based on participant responses, we offer three recommendations about how to improve health status and health care quality and access for older African Americans. While these data were obtained during the height of the COVID-19 pandemic, we find them useful and transferable to contexts beyond COVID to mitigate health care access disparities. First, we recommend that community advisory committees be formed to provide opportunities for feedback from community members and from organizations that were cited as being effective in promoting health among older African Americans. For example, churches were frequently cited as community organizations effective in reaching older African Americans and several participants recommended that churches become more involved in disseminating health information, providing health education programming and facilitating access to health care. Second, we recommend bringing access to community members. For instance, participants suggested providing mobile dental health clinics staffed by dental students to improve dental health status and mobile farmers' markets as a way to provide more fresh produce in rural communities without grocery stores. Third, we recommend examining the quality of broadband and Internet access use for older African Americans in rural communities. Even with the best of platforms, if persons either do not have quality Internet access or have experience with using technology, innovations such as telehealth will not reach communities that could greatly benefit from their use. These recommendations can assist in making needed policy enhancements to address barriers experienced by certain racial groups or socioeconomically challenged and under-resourced areas.

We acknowledge the robust data that we obtained from our sample regarding their perspectives on health disparities, access to health care, and solutions for addressing disparities. However, there are some limitations of this study that should be noted. First, the size of the sample could be perceived as a limitation. However, we used a key tenet of qualitative research, data saturation, to determine that our sample size was adequate and that collecting additional data would produce little to no new information to address our study aims. Secondly, we limited our catchment area to a specific geographic region of the State of South Carolina. While our rationale for aiming our efforts to an underserved and socioeconomically depressed part of the State were sound, this further limits our ability to generalize findings beyond comparable communities.

Several study strengths should also be noted. First, we employed an effective recruitment strategy to obtain the sample. Partnering with a local agency to focus exclusively on recruitment and data collection yielded a relatively short recruitment period (9 weeks), which allowed more time to clean, review, and analyze the data. Secondly, this study represents a growing body of work designed to understand the context of life and health during the COVID 19 pandemic. In particular, our approach to obtaining community members' perspectives about disparities in health care access during the pandemic from African American elders is one that illuminates their lived experiences. Oftentimes, researchers use quantitative data to draw conclusions about the impact of disparities on communities, without engaging community members about this topic. Other studies have observed disparities in accessing care during the COVID-19 pandemic, but few have done so by highlighting the community's voice (8–10). Lastly, this study focuses on an understudied population. Health disparities are widespread among the aging African American community, thus future research should aim to examine not only health outcomes among this group, but the key drivers of theses disparities so that culturally relevant solutions can be devised and implemented.

## Conclusion

Our qualitative approach was valuable in obtaining perspectives about access to health care during the COVID-19 pandemic from African American older adults. Our findings suggest that while traditional access to health care was not disrupted very much, telehealth efforts designed to enhance or extend health care access were challenging. Continued research with African American elders, particularly those in under-resourced communities are warranted to further elucidate these findings. Extensions of this work might include examining the underlying causes of health care access challenges, asking community members to help inform viable solutions to further improve health care access, and working alongside community members and partnering with organizations who can communicate with the population to help devise effective solutions to address and eliminate health care access issues. The wisdom and lived experience of these community members can help ensure that programs are tailored to the needs and desires of the intended audience.

## Data availability statement

The raw data supporting the conclusions of this article will be made available by the authors, without undue reservation.

## Ethics statement

Ethical review and approval was not required for the study on human participants in accordance with the local legislation and institutional requirements. Written informed consent for participation was not required for this study in accordance with the national legislation and the institutional requirements.

## Author contributions

LI conceptualized the study, conducted data analysis, and wrote the initial draft of the manuscript. CD conceptualized the study, conducted data analysis, edited, and contributed to the final draft of the manuscript. HB helped conceptualize the study, edited, and contributed to the final draft of the manuscript. YL edited, and contributed to the final draft of the manuscript. TH conducted the literature review and helped write the initial draft of the manuscript. All authors contributed to the article and approved the submitted version.

## Funding

Grant support was received from AARP South Carolina to conduct this work.

## Conflict of interest

The authors declare that the research was conducted in the absence of any commercial or financial relationships that could be construed as a potential conflict of interest.

## Publisher's note

All claims expressed in this article are solely those of the authors and do not necessarily represent those of their affiliated organizations, or those of the publisher, the editors and the reviewers. Any product that may be evaluated in this article, or claim that may be made by its manufacturer, is not guaranteed or endorsed by the publisher.
